# Pathogenic mechanisms and comprehensive therapeutic strategies of ovarian aging: targeting gut microbiota

**DOI:** 10.3389/fmicb.2026.1890866

**Published:** 2026-08-18

**Authors:** Yao Chen, Sainan Tian, Jing Zeng, Meifang Wu, Pei Tang, Lei Lei, Wen'e Liu, Li Tang

**Affiliations:** 1 The First Hospital of Hunan University of Chinese Medicine, Changsha, Hunan, China; 2 Hunan University of Chinese Medicine, Changsha, Hunan, China

**Keywords:** anti-ovarian aging, diminished ovarian reserve, gut microbiota, ovarian aging, pathogenic mechanisms, premature ovarian insufficiency, therapeutic strategies

## Abstract

Ovarian aging profoundly affects female reproductive lifespan and quality of life, and gut microbiota serves as a key regulator of reproductive aging. Accumulating studies have proven that gut microbiota dysbiosis is closely associated with ovarian aging, and restoring disturbed gut microbiota can effectively delay this process. Nevertheless, most current researches focus on a single subtype of ovarian aging, and relevant targeted interventions have not been systematically summarized. This review elaborates the alterations of gut microbiota during physiological and pathological ovarian aging, and explores the core mechanisms by which microbiota dysbiosis drives ovarian aging, including dysfunction of the “estrobolome,” deficiency of short-chain fatty acids (SCFAs), abnormal metabolism of bile acids (BAs) and tryptophan (TRP), as well as chronic low-grade inflammation caused by intestinal barrier impairment. We also comprehensively summarize gut microbiota-targeted prevention and treatment strategies, including conventional approaches such as probiotics, prebiotics, synbiotics, fecal microbiota transplantation (FMT), metabolite supplementation and Chinese herbal medicines, as well as emerging therapies like stem cell therapy, extracellular vesicle and exosome therapy. In addition, we discuss the limitations of existing studies and challenges in clinical translation, and prospect future research directions including multi-omics analysis and precise clinical intervention. This review aims to provide a theoretical basis for developing novel strategies to retard ovarian aging and improve female reproductive health.

## 1 Introduction

Ovarian aging is a progressive and irreversible biological process that drives the decline of female reproductive competence and deterioration of endocrine health (Chen et al., 2026a; Liu et al., 2025c; Walter, 2025), and serves as the pathological foundation for long-term complications including cardiovascular diseases, neurodegenerative diseases and postmenopausal osteoporosis (Liu et al., 2025c; Rocca et al., 2018). Conventional mechanistic studies have clarified that ovarian aging is essentially characterized by the progressive decline in follicle quantity and quality, which involves the crosstalk of multiple pathways such as excessive activation and apoptosis of primordial follicles, accumulated oxidative stress, mitochondrial DNA damage and autophagic dysfunction, telomere shortening, epigenetic reprogramming and ovarian microenvironmental fibrosis (Park et al., 2021; Wang et al., 2023; Zhu et al., 2022). Nevertheless, these factors alone cannot fully explain the interindividual heterogeneity and temporal discrepancy of ovarian aging, and novel regulatory mechanisms are urgently required to meet clinical demands.

In recent years, the concept of the gut-ovary axis has gained growing attention, and bidirectional interactions between gut microbiota and ovarian function have been validated. As complex and dynamic microbial communities colonizing the gastrointestinal tract, gut microbiota play pivotal roles in regulating host metabolism, immune homeostasis and endocrine function (Luo et al., 2024; Wang et al., 2024). Accumulating evidence from animal models and human clinical studies indicates that remarkable structural and functional alterations occur in gut microbiota during ovarian aging (Huang et al., 2024; Lyu et al., 2025). Causal studies have demonstrated that gut microbiota dysbiosis is not merely a consequence, but also a core driving factor of ovarian aging (Wen et al., 2024). Restoring dysregulated gut microbiota can effectively delay ovarian aging. Most existing studies only focus on a single type of ovarian aging, and current targeted therapies are largely limited to probiotic supplementation and fecal microbiota transplantation, without a systematic and comprehensive overview of diverse intervention strategies.

Accordingly, this review comprehensively summarizes the latest advances in research on gut microbiota and ovarian aging. It systematically describes the characteristics of gut microbiota dysbiosis during physiological and pathological ovarian aging, and elucidates how gut microbiota regulates ovarian function via the estrogen–gut microbiota axis, metabolite-related signaling pathways and intestinal barrier integrity. We also summarize diverse microbiota-targeted therapeutic strategies and their clinical translational potential. Furthermore, we discuss the major limitations of current studies and the challenges in translational medicine, and highlight future research directions including multi-omics analysis, precise intervention and clinical application. This work aims to lay a solid theoretical foundation for developing novel interventions to delay ovarian aging and improve female reproductive health.

## 2 Ovarian aging

Ovarian aging represents a hallmark process of female reproductive functional decline, which is not a single terminal event but a continuous spectrum ranging from normal physiological function to complete functional deterioration (Hirano et al., 2025). It can be classified into physiological ovarian aging and pathological ovarian aging according to etiological factors and onset time (Wang et al., 2023). Physiological ovarian aging refers to the naturally occurring decline of endocrine and reproductive functions in women over 40 years old (Cavalcante et al., 2023). Pathological ovarian aging is defined as the premature deterioration of ovarian function prior to the expected age induced by genetic, immune, iatrogenic, or environmental factors (Evangelinakis et al., 2024; Isola et al., 2024; Lopez et al., 2023), mainly including diminished ovarian reserve (DOR) and premature ovarian insufficiency (POI). DOR is manifested by reduced quantity and/or impaired quality of oocytes, decreased serum anti-Müllerian hormone (AMH) level, declined antral follicle count and elevated follicle-stimulating hormone (FSH), accompanied by impaired fertility (Annalisa et al., 2022). POI is diagnosed as menstrual irregularities lasting for 4 consecutive months in women younger than 40 years old, with basal serum FSH levels exceeding 25 IU/L. In addition to aggravated hypoestrogenic symptoms, POI contributes to infertility, severe bone loss and premature cardiovascular events (European et al., 2016). Premature ovarian failure (POF) represents the end-stage of POI (Bricaire et al., 2013).

## 3 Gut microbiota

Gut microbiota refers to the entire microbial communities colonized in the human gastrointestinal tract, encompassing bacteria, archaea, fungi and viruses, among which bacteria constitute the predominant component with more than 1,000 identified species. The total number of microbial genes is approximately 150 times that of the human genome, hence termed the human “second genome.” *Firmicutes* and *Bacteroidetes* serve as the dominant phyla, and their community composition is modulated by heredity, age, dietary patterns, and medications to maintain dynamic homeostasis under physiological conditions (de Vos et al., 2022; Makki et al., 2018; Qin et al., 2010). The core biological functions of gut microbiota include fermenting dietary fiber to produce SCFAs that supply energy for intestinal epithelial cells and regulate immune responses (Li et al., 2022), metabolizing TRP to generate indole derivatives (Agus et al., 2018), converting primary BAs into secondary BAs to regulate host lipid metabolism and energy homeostasis (Su et al., 2023), and maintaining intestinal mechanical and immune barriers via competitive colonization resistance, antimicrobial substance secretion and modulation of intestinal epithelial tight junctions (Takiishi et al., 2017).

## 4 Bidirectional regulation between ovarian aging and gut microbiota

### 4.1 Ovarian aging remodels the diversity, species abundance, and function of gut microbiota

The progression of ovarian aging drives the succession of gut microbiota from a homeostatic state to a dysbiotic state, and accumulating studies have uncovered the complex and profound association between alterations in gut microbial diversity and female ovarian aging. As shown in Table 1, cross-sectional data reported by Zhao et al. (2019) demonstrate that postmenopausal women exhibit significantly reduced gut microbial alpha diversity, marked depletion of *Firmicutes* and *Roseburia*, and overrepresentation of *Bacteroidetes* and *Tolumonas*, a harmful bacterium closely correlated with decreased bone density. Santos-Marcos et al. (2018) revealed that the overall gut microbial community profile in postmenopausal women loses female-specific characteristics and shifts toward a male microbial phenotype. Specifically, the *Firmicutes*/*Bacteroidetes* ratio and the abundance of *Roseburia* and *Lachnospira* in postmenopausal women are comparable to those in male controls matched for age, body mass index (BMI) and nutritional background, but significantly lower than those in premenopausal women. Meanwhile, the abundances of *Prevotella, Parabacteroides* and *Bilophila* are markedly elevated, accompanied by a metabolic profile prone to producing pro-inflammatory and metabolism-disrupting metabolites. Wang J. et al. (2023) identified that intestinal *Eubacterium hallii* and *Eubacterium ventriosum* are significantly and protectively correlated with POI; conversely, *Enterobacter* and *Terrisporobacter* are associated with adverse POI-related alterations. Wu et al. (2021) compared gut microbial features between 35 POI patients and 18 healthy females and found significant changes in gut microbial beta diversity rather than alpha diversity in POI patients. In terms of microbial composition, POI patients present reduced abundance of beneficial bacteria and increased enrichment of opportunistic pathogens. Correlation analyses indicate that gut microbiome alterations in POI women are strongly associated with serum levels of FSH, luteinizing hormone (LH), estradiol (E_2_), AMH, as well as the FSH/LH ratio. Jiang et al. (2021) reported no significant differences in gut microbial alpha diversity among healthy controls, untreated POI patients and POI patients receiving hormone replacement therapy, whereas POI patient's exhibit decreased gut microbial beta diversity. These findings suggest that gut microbiota dysbiosis in POI is primarily characterized by aberrant abundance of specific genera rather than a decline in overall microbial diversity, leading to homogenization of the gut microecology and loss of natural microbial variation observed in healthy individuals. With regard to the end-stage of POI, Zhang et al. (2025c) demonstrated that the patients show pronounced reductions in the abundance of *Lactobacillus* and *Bifidobacterium*, accompanied by a sharp decrease in β-glucuronidase (gmGUS) activity, and the abundance of these two microbial genera is positively correlated with gmGUS activity. Importantly, such microbial and enzymatic alterations are closely correlated with decreased AMH and E_2_ levels, as well as elevated FSH and LH levels. Further analyses validated that the combined diagnostic model integrating gut microbiota and enzymatic activity exhibits excellent diagnostic efficiency, with an Area Under the ROC Curve (AUC) of up to 0.912, providing a novel non-invasive strategy for early screening. Nevertheless, this study fails to establish a causal relationship between gut microbial alterations and the end-stage of POI progression. Additionally, it only detects limited microbial taxa, cannot fully reflect overall structural changes of the gut microbiota, and is constrained by a small sample size. Notably, systematic case-control studies focusing on gut microbial characteristics in DOR patients remain unreported. Large-scale, multicenter, prospective clinical case-control studies integrating metagenomic sequencing with serum and fecal metabolomics are urgently required to fill the research gap regarding the taxonomic composition, functional pathways and metabolic profiles of gut microbiota in DOR patients. Gut microbiota dysbiosis induces the occurrence of ovarian aging.

**Table 1:** Human cohort studies on the association between ovarian aging and the gut microbiota.

Types	Study subjects	Changes in gut microbiota diversity and composition	Functional alterations of the gut microbiota	Correlation analysis
Menopause (Zhao et al., 2019)	24 premenopausal women and 24 postmenopausal women	Postmenopausal women exhibit significantly reduced α-diversity, decreased abundance of *Firmicutes* and *Roseburia*, increased abundance of *Bacteroidetes* and *Tolumonas*, and a lower *Firmicutes/Bacteroidetes* ratio. Butyrate-producing core genera, including *Lachnospira, Roseburia, Faecalibacterium*, and *Parabacteroides*, are enriched in premenopausal women	Reduced steroid hormone metabolic capacity and enhanced lipopolysaccharide biosynthesis pathways are observed after menopause	Serum E_2_ levels are positively correlated with *Roseburia* and *Lachnospira* and negatively correlated with *Sutterella* and *Bilophila*. FSH levels are significantly negatively correlated with the abundance of butyrate-producing genera
Menopause (Santos-Marcos et al., 2018)	17 premenopausal women and 19 age-matched men; 20 postmenopausal women and 20 age-matched men	No significant difference in α-diversity is observed. Premenopausal women exhibit a higher *Firmicutes/Bacteroidetes* ratio and greater relative abundances of *Lachnospira* and *Roseburia*, whereas the abundances of *Prevotella, Parabacteroides*, and *Bilophila* are lower than those in postmenopausal women	Estrogen-associated steroid metabolic pathways are attenuated after menopause, and the gut microbiota shifts toward a pro-inflammatory phenotype	Serum E_2_ levels are positively correlated with the abundances of *Roseburia* and *Lachnospira*
POI (Wang J. et al., 2023)	A total of 13,266 participants from the MiBioGen consortium and 424 POI cases with 181,796 controls from the FinnGen consortium (R8)	/	/	Higher abundances of the *Eubacterium hallii* group and *Eubacterium ventriosum* group are associated with a reduced risk of POI, whereas increased abundances of *Intestinibacter* and *Terrisporobacter* are associated with an increased risk of POI
POI (Wu et al., 2021)	18 healthy controls and 35 patients with POI	Patients with POI exhibit altered β-diversity without significant changes in overall microbial diversity. The phylum *Firmicutes* and the genera *Bulleidia* and *Faecalibacterium* are more abundant in healthy women, whereas the phylum *Bacteroidetes* and the genera *Butyricimonas, Dorea, Lachnobacterium*, and *Sutterella* are significantly enriched in women with POI	Autoimmune- and chronic inflammation-related pathways are activated	*Firmicutes* and *Faecalibacterium* are positively correlated with serum E_2_ and AMH levels and negatively correlated with FSH levels and the FSH/LH ratio
POI (Jiang et al., 2021)	10 healthy women, 10 patients with hormone replacement therapy (HRT)-treated POI, and 10 patients with untreated POI	No significant difference in α-diversity is observed among the three groups, whereas β-diversity is reduced in patients with POI. The abundance of the genus *Eggerthella* is significantly increased in patients with POI	Ovarian fibrosis is increased	The abundance of *Eggerthella* is associated with systemic metabolism and the progression of ovarian fibrosis
The end-stage of POI (Zhang et al., 2025c)	62 patients with the end-stage of POI and 52 healthy controls	Patients with the end-stage of POI exhibit lower abundances of *Lactobacillus, Bifidobacterium*, and gut microbial β-glucuronidase (gmGUS) than healthy controls.	/	gmGUS is positively correlated with serum E2 levels and negatively correlated with AMH, FSH, and LH levels

### 4.2 Gut microbiota dysbiosis initiates ovarian aging

The gut-ovary axis constitutes a pivotal pathway governing reproductive aging. Gut dysbiosis is primarily characterized by reduced microbial diversity, alongside shifts in species abundance and function. The gut microbiota modulates ovarian aging via three core mechanisms: endocrine disruption, metabolic regulation, and chronic low-grade inflammation. First, the gut microbiota regulates enterohepatic circulation of estrogen. Dysbiosis disturbs estrogen metabolism and sex hormone secretion, impairs hypothalamic-pituitary-ovarian axis function, and accelerates ovarian aging. Second, the gut microbiota participates in host synthesis of short-chain fatty acids as well as bile acid and tryptophan metabolism. Imbalanced microbiota reduces short-chain fatty acid production and disrupts bile acid and tryptophan metabolism, triggering metabolic anomalies in ovarian tissue, suppressing follicular proliferation, promoting granulosa cell apoptosis, and directly depleting ovarian reserve. Meanwhile, dysbiosis compromises intestinal barrier integrity, facilitates endotoxin translocation into circulation, activates the systemic and ovarian local NF-κB inflammatory cascade, sustains chronic low-grade ovarian inflammation, and leads to follicular atresia and ovarian microenvironmental fibrosis. Details are presented as follows.

#### 4.2.1 Functional deficiency of the “estrobolome” leads to further depletion of circulating estrogens and accelerates ovarian aging

Reduced estrogen levels represent a direct manifestation of ovarian aging (Shi et al., 2026). Gut microbiota are involved in the synthesis and metabolism of estrogens (Wang et al., 2026). The “estrobolome” refers to the collective pool of bacterial genes encoding enzymes such as gmGUS and β-glucosidase in intestinal microorganisms. It functions to dissociate and reactivate estrogens, participate in the enterohepatic circulation of estrogens and modulate their bioavailability (Kumari et al., 2024). Estrogens synthesized in the ovaries are efficiently recycled and reused through reactivation mediated by the “estrobolome” (Baker et al., 2017). Additionally, the “estrobolome” maintains circulating estrogen concentrations by regulating the expression of estrogen synthesis-related enzymes in peripheral tissues (Chen Q. et al., 2024).

The liver is the primary organ responsible for estrogen metabolic inactivation. Estrogens undergo phase II metabolic reactions in the liver and conjugate with glucuronic acid to form inactive estrogen-glucuronide complexes, which lose the capacity to bind estrogen receptors and are subsequently excreted into the intestinal tract along with Bas (Stanczyk, 2024). Specific intestinal microbiota encode and secrete gmGUS to hydrolyze estrogen-glucuronide conjugates and release bioactive free estrogens. The activated free estrogens are then reabsorbed across the intestinal epithelium into the hepatic portal circulation and enter systemic blood circulation to exert biological functions (Wang et al., 2025). This physiological process is defined as the enterohepatic circulation of estrogens, which enables efficient estrogen recycling, with gmGUS acting as a core functional mediator (Hu et al., 2023). Chaudhary et al. (2026) demonstrated that estrogen deficiency not only reshapes gut microbial composition, but also markedly suppresses gmGUS activity and downregulates GUSB gene expression. These findings indicate that estrogen insufficiency triggers gut microbiota dysbiosis and impairs the estrogen-activating capacity of the “estrobolome,” thereby reducing the quantity of estrogens reabsorbed via enterohepatic circulation and further lowering circulating estrogen levels, ultimately forming a self-amplifying vicious positive feedback loop. Another clinical study (Martínez-Nortes et al., 2026) validated that postmenopausal women exhibit decreased abundance of gmGUS-producing beneficial bacteria in the gut, which diminishes intestinal estrogen reabsorption efficiency and weakens systemic recycling capacity of limited endogenous estrogen resources.

Gut microbiota also modulated circulating estrogen concentrations by regulating estrogen synthetases in adipose tissues. Cytochrome P450 Family 19 Subfamily A Member 1 (CYP19A1), namely aromatase, is the rate-limiting enzyme catalyzing the conversion of androgens to estrogens. It is highly expressed in adipose tissues and constitutes the major source of endogenous estrogens in postmenopausal females (Tüzüner et al., 2016). Accumulating evidence reveals that estrogen deficiency accompanied by gut microbiota disturbance inhibits CYP19 expression in adipose tissues and blunts the compensatory estrogen supplementation capacity of peripheral tissues (Chen Q. et al., 2024). Supplementation with *Lactobacillus gasseri CCFM1255* significantly upregulates adipose CYP19 expression and promotes peripheral estrogen synthesis by remodeling gut microbial structure and reshaping serum metabolic profiles (Chen Q. et al., 2024).

Collectively, gut microbiota serve as essential regulators for maintaining estrogen homeostasis. Gut microbiota dysbiosis not only compromises gmGUS-dependent enterohepatic circulation of estrogens but also represses estrogen biosynthesis in peripheral tissues, reduces estrogen bioavailability at multiple levels, and consequently exacerbates persistent estrogen deficiency to facilitate the progression of ovarian aging.

#### 4.2.2 Deficiency of gut microbiota-derived SCFAs mediates granulosa cell apoptosis and follicular atresia to promote ovarian aging

SCFAs, mainly including acetate, propionate and butyrate, are primary metabolic end-products derived from intestinal microbial fermentation of dietary fibers (Ahmad et al., 2025). The concentration of SCFAs reaches approximately 150 μmol/L in ovarian follicular fluid, implying their direct local regulatory roles within ovarian tissues (Xu et al., 2023). Gut microbiota dysbiosis triggers reduced abundance or diversity of SCFAs-producing microbes, resulting in systemic and local SCFAs insufficiency, which mediates ovarian granulosa cell apoptosis and follicular atresia, thereby facilitating ovarian aging (Herndez-Acosta et al., 2025). Butyrate directly acts on ovarian granulosa cells via G protein-coupled receptor 41 (GPR41) and G protein-coupled receptor 43 (GPR43) (Ye et al., 2021). GPR43 is ubiquitously expressed in granulosa cells and theca cells. SCFAs depletion weakens GPR43 signaling, suppresses the activity of the phosphatidylinositol 3-kinase/protein kinase B (PI3K/Akt) pathway, downregulates the expression of anti-apoptotic B-cell lymphoma-2 (Bcl-2), and upregulates pro-apoptotic mediators including Bcl-2-associated X protein (Bax) and cleaved caspase-3, thereby accelerating follicular atresia and granulosa cell apoptosis. Propionate activates GPR43 in periovarian white adipose tissues to stimulate leptin secretion, which further restrains granulosa cell apoptosis, alleviates follicular atresia and promotes follicular maturation (Xu et al., 2025). The size of the primordial follicle pool determines female reproductive lifespan. Munyoki et al. (2025) reported that germ-free mice undergo accelerated primordial follicle loss, increased ovarian collagen deposition and prominently shortened reproductive lifespan during the weaning transition period, a critical stage for gut microbial transition from milk-associated flora to solid diet-associated flora. Exogenous SCFAs supplementation effectively ameliorated ovarian dysfunction in germ-free mice, confirming that gut microbiota sustained primordial follicle pool homeostasis and extend reproductive lifespan in female mammals via SCFAs-mediated metabolic signaling cascades.

Ovarian aging is frequently accompanied by inflammaging (Franceschi et al., 2000; Zeng et al., 2024). SCFAs are essential for preserving intestinal barrier integrity and mitigating systemic and local chronic inflammation. As the predominant energy substrate for colonic epithelial cells, butyrate upregulates the expression of tight junction proteins including Occludin, Claudin-1 and Zonula Occludens-1 (ZO-1), and exerts potent anti-inflammatory effects (Encarnação et al., 2015; Vital et al., 2014). SCFAs deficiency disrupts intestinal barrier function and facilitates Lipopolysaccharide (LPS) translocation into portal circulation, triggering chronic low-grade endotoxemia. Translocated LPS further activates the ovarian Toll-like receptor 4/nuclear factor kappa-B (TLR4/NF-κB) signaling pathway, promotes the secretion of pro-inflammatory cytokines such as IL-6 and tumor necrosis factor-α (TNF-α), elevates oxidative stress levels, and ultimately accelerates follicular atresia and induces oocyte apoptosis (Adhikari et al., 2025; Liu et al., 2026).

Taken together, gut microbiota-derived SCFAs constitute crucial metabolic mediators for sustaining ovarian homeostasis. Reduced abundance of SCFAs-producing bacteria leads to insufficient SCFAs synthesis, which not only attenuates follicular supportive signals to promote granulosa cell apoptosis and primordial follicle pool exhaustion, but also disrupts intestinal barrier integrity, augments LPS translocation and activates the TLR4/NF-κB inflammatory cascade, thereby triggering chronic low-grade inflammation and further accelerating follicular atresia and ovarian functional decline. Accordingly, dysregulation of the gut microbiota-SCFAs axis facilitates ovarian aging via multiple mechanisms encompassing metabolic modulation, barrier impairment, and inflammation-mediated pathways.

#### 4.2.3 Gut microbiota-mediated BAs dysmetabolism drives follicle depletion and ovarian aging

BAs are catabolic derivatives of cholesterol and act as multifunctional metabolic regulators with extensive signaling activities (Cai et al., 2022; Sah et al., 2022). Primary BAs including cholic acid (CA) and chenodeoxycholic acid (CDCA) are synthesized in the liver (Fleishman and Kumar, 2024; Guo et al., 2024). After conjugation with glycine or taurine, primary BAs are secreted into the intestinal tract with bile. Under the catalysis of intestinal microbial bile salt hydrolase (BSH) and 7α-dehydroxylase, conjugated primary BAs undergo deconjugation and dehydroxylation reactions to generate secondary BAs, such as deoxycholic acid (DCA), lithocholic acid (LCA), and ursodeoxycholic acid (UDCA). These metabolites reach ovarian tissues through enterohepatic circulation or systemic circulation (Jia et al., 2024, 2018). Ovarian farnesoid X receptor (FXR) and Takeda G protein-coupled receptor 5 (TGR5) sense alterations in circulating bile acid concentration and composition, and transduce these signals to regulate follicular development and steroid hormone synthesis (Takae et al., 2019; Zhao et al., 2025). Gut microbiota dysbiosis may alter the synthesis, biotransformation and circulation of BAs to induce aberrant BAs profiles, which further disrupts signal transduction mediated by BAs-sensing receptors including FXR and TGR5, disturbs follicle quiescence, granulosa cell survival and steroidogenesis, and drives the initiation and progression of ovarian aging.

BAs are indispensable for maintaining ovarian microenvironmental homeostasis, and aberrant bile acid profiles trigger ovarian functional decline and ovarian tissue damage. In mouse models with circadian rhythm disturbance-induced impaired oocyte quality, the abundance of *Clostridium* and *Bacteroides* (key genera involved in secondary bile acid biosynthesis) is decreased, accompanied by reduced fecal levels of LCA, NorDCA, THDCA, isoalloLCA, 7-KetoLCA and CDCA. Exogenous supplementation with LCA or NorDCA efficiently improves oocyte quality and early embryonic developmental potential, and 30 mg/kg LCA also reverses the decline in total intestinal bile acid concentrations (Li et al., 2023). Nevertheless, excessive accumulation of DCA and LCA exerts prominent cytotoxic effects. Maternal vinylcyclohexene dioxide (VCD) exposure increases the abundance of intestinal Parabacteroides and Flexispira (microorganisms responsible for secondary bile acid synthesis) in offspring mice, accompanied by markedly elevated levels of secondary BAs including NorDCA, LCA-3S and DCA, which are closely correlated with impaired ovarian function in progeny (Li et al., 2024). Wei et al. (2022) detected significantly elevated glycodeoxycholic acid (GDCA) levels and decreased free bile acid contents in atretic buffalo follicles, and verified that GDCA promotes granulosa cell apoptosis and suppresses steroid hormone secretion. Treatment with 2,500 nmol/L glycochenodeoxycholic acid disrupts plasma membrane integrity in KGN cells and increases lactate dehydrogenase (LDH) release, whereas taurocholic acid and glycocholic acid fail to induce LDH leakage, confirming distinct cytotoxicity among BAs with different chemical structures (Avcioglu and Berkel, 2026).

As a nuclear receptor transcription factor, FXR in granulosa cells can be activated by multiple BAs including CA, CDCA and DCA (Perino et al., 2021). FXR inhibits local inflammatory cascades via antagonizing the NF-κB signaling pathway (Chávez-Talavera et al., 2017). Moreover, FXR directly binds to the promoter region of the Forkhead box O3 (FoxO3) gene to maintain primordial follicle quiescence, precisely control the consumption rate of ovarian reserve and prevent premature ovarian reserve exhaustion. FXR knockout mice exhibit excessive primordial follicle activation and impaired ovarian reserve capacity (Tomioka et al., 2026). Notably, aberrant overactivation of FXR signaling also causes ovarian functional impairment. Dietary CA administration induces abnormal hyperactivation of ovarian FXR signaling, suppresses steroid hormone synthesis pathways, and leads to reduced ovarian weight, disturbed estrous cycles, decreased antral follicle and corpus luteum numbers, as well as declined serum progesterone (P) and E_2_ levels in mice (Zhu et al., 2025). Another study revealed that Lactobacillus salivarius reduces tauoursodeoxycholic acid (TUDCA) levels by modulating BSH activity, which relieves the inhibitory effect of TUDCA on intestinal FXR and triggers excessive intestinal FXR activation. This process further inhibits the production of downstream protective factor interleukin-22 (IL-22) and accelerates granulosa cell death (Zhao et al., 2026).

TGR5 is a plasma membrane-localized G protein-coupled receptor. Upon bile acid-mediated activation, TGR5 initiates the cyclic adenosine monophosphate/protein kinase A (cAMP/PKA) pathway to enhance cellular energy metabolism and antioxidant defense, and modulates steroid hormone synthesis via regulating stromal-epithelial intercellular communication (Fleishman and Kumar, 2024). Functional studies have confirmed that CDCA markedly upregulates the expression of steroidogenic acute regulatory protein (StAR) and cholesterol side-chain cleavage enzyme (CYP11A1) in ovarian tissues through TGR5 activation, facilitates ovarian steroidogenesis and improves embryo implantation efficiency (Chen M. et al., 2024). TUDCA alleviates endoplasmic reticulum stress and oxidative stress induced by high-glucose conditions in porcine embryonic cells via activating TGR5 signaling, promotes DNA damage repair, and consequently elevates embryonic survival rate and developmental competence. Blockade of TGR5 signaling completely abolishes these beneficial effects of TUDCA (Dicks et al., 2021). Emerging evidence indicates that hypothalamic TGR5 perceives dynamic changes in BAs profiles and modulates follicular development and ovulation by regulating kisspeptin signaling and gonadotropin-releasing hormone (GnRH) secretion (Vanden Brink et al., 2024).

#### 4.2.4 Gut microbiota-mediated imbalanced TRP metabolism promotes inflammation and oxidative stress to drive ovarian aging progression

TRP is an essential amino acid exclusively acquired from dietary sources. It is primarily catabolized via three major pathways: the kynurenine (KYN) pathway, the serotonin pathway and the indole pathway. TRP metabolites exert core regulatory functions in maintaining host immune homeostasis, redox balance and endocrine stability (Holeček, 2026). During ovarian aging, gut microbiota dysbiosis-induced TRP metabolic disturbance triggers ovarian inflammation and oxidative stress via aberrant KYN pathway activation and depletion of indole-derived metabolites, impairs granulosa cell function and follicular development, and accelerates ovarian functional deterioration.

Approximately 95% of systemic TRP is catalyzed by indoleamine 2,3-dioxygenase (IDO) and tryptophan 2,3-dioxygenase (TDO) to generate KYN, which is further degraded into nicotinamide adenine dinucleotide (NAD+) and kynurenic acid (Ou et al., 2025). Accumulation of pro-inflammatory pathogenic intestinal microbiota and their metabolic products induced local intestinal and systemic chronic low-grade inflammation (Selvakumar and Samsudin, 2025). Shen et al. (2023) verified that LPS significantly elevates IDO activity, accelerates TRP degradation in ovarian tissues and causes abnormal KYN accumulation. Excessive KYN directly disturbs steroid hormone synthesis in theca cells and granulosa cells, resulting in ovulatory dysfunction and impaired reproductive performance. Oxidative stress disrupts gut microbial diversity, triggers hyperactivation of the KYN pathway to deplete endogenous TRP reserves, inhibits melatonin biosynthesis and weakens antioxidant capacity, ultimately inducing follicular atresia and ovarian cell apoptosis in laying hens (Wang et al., 2021). Therefore, excessive activation of the KYN pathway causes ovarian functional decline and promotes ovarian aging via exacerbating oxidative stress and inflammatory responses. Intriguingly, 100 μmol/L L-KYN enhances antioxidant and anti-apoptotic capacities of bovine oocytes, and improves oocyte maturation rate and subsequent embryonic developmental competence (Lu et al., 2025). However, clinical investigations yield contradictory results against the above preclinical findings. Both IDO protein and mRNA expression levels are significantly downregulated in ovarian granulosa cells of patients with POI compared with healthy controls (Hu A. et al., 2025). Reduced IDO expression blocks TRP to KYN conversion and suppresses downstream NAD+ biosynthesis (Di Emidio et al., 2024). As a pivotal cofactor involved in cellular energy metabolism and DNA damage repair, decreased NAD+ levels impair energy supply and genomic stability in granulosa cells and oocytes, thereby expediting follicle depletion (Yang et al., 2023). Collectively, these studies illustrate that both excessive activation and suppression of the TRP-KYN pathway impair ovarian function through distinct molecular mechanisms, and only physiological balanced TRP metabolism exerts protective effects on ovarian tissues.

A fraction of intestinal TRP is directly metabolized into diverse indole derivatives by commensal gut bacteria, among which indole-3-propionic acid (IPA), indole-3-acetic acid (IAA), indole-3-lactic acid (ILA), indole-3-carboxaldehyde (IAld), and indole-3-carbinol (I3C) are well-characterized metabolites (Liu et al., 2023; Moroni, 1999; Zhang et al., 2026). These indole derivatives function as endogenous ligands of the aryl hydrocarbon receptor (AhR). AhR participates in multiple physiological processes including ovarian folliculogenesis, steroid hormone synthesis, embryo implantation and pregnancy maintenance, and plays a vital role in sustaining normal ovarian function and regulating reproductive lifespan (Dean and Flaws, 2026; Hernández-Ochoa et al., 2009; Huang et al., 2025b). Untargeted metabolomic profiling reveals that follicular fluid TRP and indole metabolite levels are significantly reduced in DOR patients relative to subjects with normal ovarian reserve (Liu et al., 2023). Deficiency of indole metabolites attenuates AhR-mediated ovarian protective signaling, and exogenous IPA and IAA supplementation alleviates bone loss and preserves intestinal barrier integrity in ovariectomized mice in an AhR-dependent manner (Chen C. et al., 2024).

#### 4.2.5 Gut microbiota dysbiosis accelerates ovarian aging via disruption of intestinal barrier integrity and induction of inflammatory responses

The intestinal barrier is a core structural foundation maintaining systemic metabolic and immune homeostasis. Intestinal epithelial cells, tight junction proteins, mucus layers, and mucosal immune systems collectively constitute the mechanical, chemical, and immunological barriers of the intestine (Selvakumar and Samsudin, 2025). Under gut microbiota dysbiosis conditions, disrupted intercellular tight junctions increase intestinal permeability and trigger intestinal leakage, allowing massive translocation of intestinal harmful substances such as bacterial endotoxins into systemic circulation. This process initiates persistent systemic chronic low-grade inflammation and accelerates ovarian functional decline via multiple downstream signaling cascades (Escalante et al., 2025).

Intercellular tight junctions between intestinal epithelial cells constitute the core mechanical intestinal barrier, which is mainly composed of transmembrane proteins (Claudin, Occludin) and cytoplasmic scaffolding protein ZO-1 (Fu et al., 2025). *Lactobacillus* maintains intestinal epithelial homeostasis via secreting lactic acid and bacteriocins (Di Cerbo et al., 2016), while *Akkermansia* contributes to intestinal mucus layer formation and tight junction protein stabilization (Xu et al., 2025). Overgrowth of opportunistic pathogens such as Enterobacteriaceae and Desulfovibrio secretes proteases and toxic metabolites that directly destruct intestinal tight junction structures (Guignot et al., 2007; Nie et al., 2022). Long-term sucralose exposure induces gut microbiota dysbiosis, disrupts intestinal tight junctions and elevates LPS leakage in mice. Elevated circulating LPS activates the TLR4/NF-κB pathway in ovarian granulosa cells, promotes the release of pro-inflammatory cytokines including IL-1β, IL-6 and TNF-α, and facilitates ovarian aging and ovarian dysfunction (Chen et al., 2026b). Gut microbial-derived SCFAs are crucial for maintaining the integrity of intestinal epithelial tight junctions. In particular, butyrate preserves intestinal barrier integrity and alleviates systemic inflammation by activating intestinal epithelial GPR41 and GPR43, inhibiting histone deacetylase activity and upregulating the expression of ZO-1, Claudin and Occludin (Fu et al., 2025). SCFAs deficiency compromises intestinal barrier integrity, facilitates LPS entry into systemic circulation to trigger widespread inflammation, directly induces granulosa cell apoptosis, exacerbates local ovarian inflammation and oxidative stress, and ultimately accelerates follicle depletion (Archana et al., 2024; Mukhopadhya and Louis, 2025). In summary, impaired intestinal barrier establishes a sustained low-grade inflammatory microenvironment favoring ovarian aging. On the one hand, translocated LPS initiates direct ovarian injury via the TLR4 signaling cascade; on the other hand, insufficient protective metabolites represented by SCFAs aggravate ovarian damage. These two pathological factors synergistically promote follicular atresia and progressive exhaustion of ovarian reserve.

**Figure 1:**
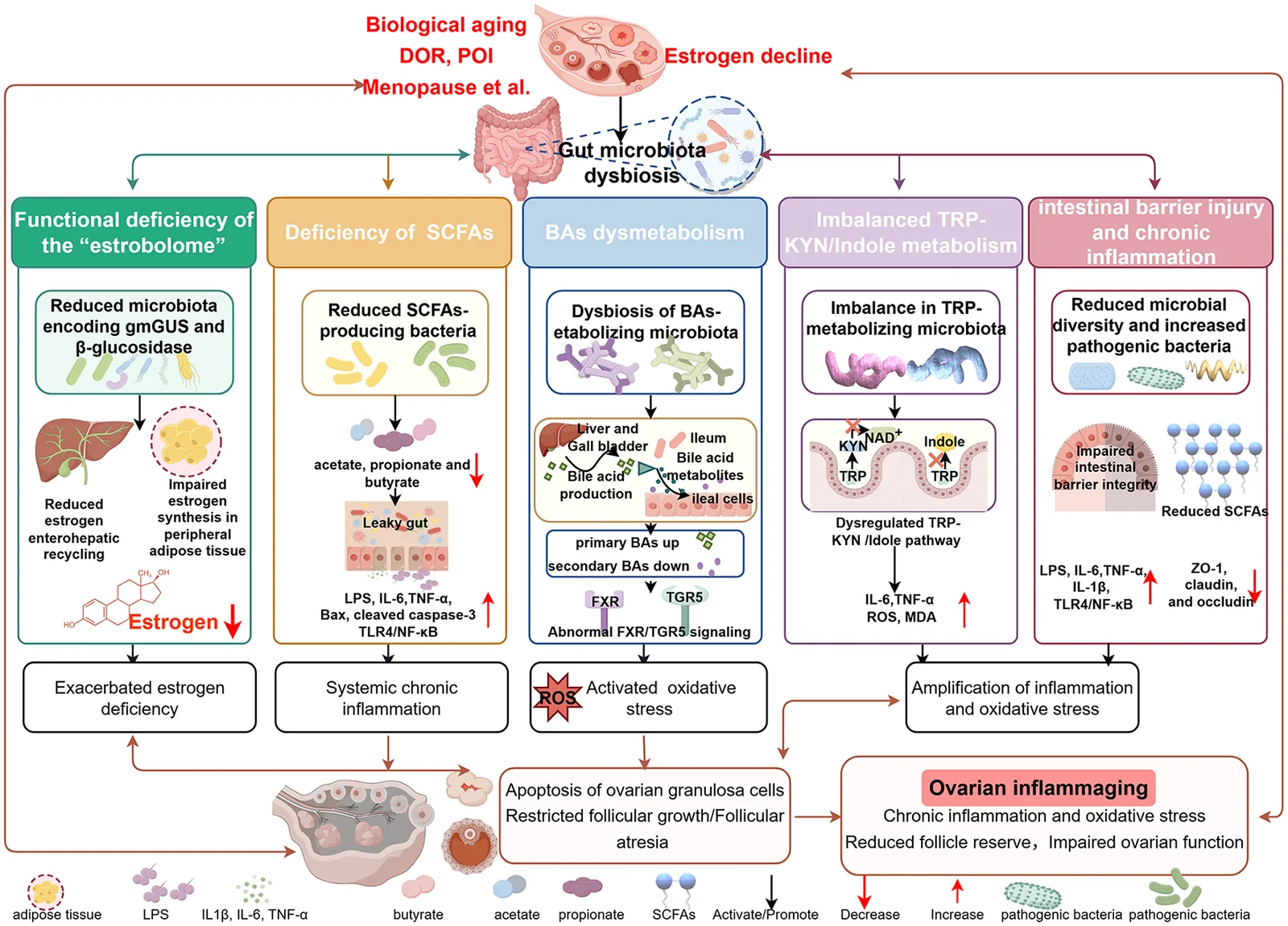
Pathological network of bidirectional modulation between ovarian aging and gut dysbiosis.

In summary, ovarian aging and gut microbiota interact bidirectionally. In patients with different ovarian dysfunctions, ovarian aging remodels gut microbial structure and host metabolic phenotypes, while gut dysbiosis triggers multiple pathological cascades to promote ovarian aging (Figure 1). Notably, gut microbiota exert regulatory effects on ovarian function independent of direct cellular crosstalk. Instead, microbial metabolic reprogramming acts as the core mediating mechanism that bridges systemic metabolic dysregulation with local molecular aberrations in ovarian tissues, constituting a pivotal upstream regulatory hub for the initiation and progression of ovarian aging. Gut microbial dysbiosis profoundly modulates the circulating abundance of microbial-derived metabolites, such as SCFAs, BAs, and TRY catabolites, as well as LPS. These peripheral bioactive metabolites are absorbed via the intestinal epithelium and enter the systemic circulation, thereby remotely targeting ovarian tissues. They further modulate key pathological molecular cascades, including granulosa cell apoptosis, excessive follicular atresia, ovarian oxidative stress, and chronic inflammatory activation, ultimately leading to diminished ovarian reserve, disrupted sex hormone homeostasis, and deteriorated ovarian microenvironment. From the perspective of mechanistic hierarchy, gut microbial metabolic dysfunction serves as the primary initiating trigger, whereas ovarian inflammation, cellular apoptosis, and oxidative stress represent subsequent downstream molecular effector responses. Microbial metabolites function as indispensable circulating signaling mediators that connect intestinal microecological homeostasis with ovarian physiological function. This mechanistic paradigm fundamentally explains the central dominance of metabolic pathways in gut microbiota-targeted interventions against ovarian aging.

## 5 Therapeutic strategies targeting gut microbiota and related metabolites for the prevention and treatment of ovarian aging

### 5.1 Supplementation of probiotics, prebiotics, and synbiotics

As viable beneficial microorganisms, probiotics exhibit great potential in ameliorating ovarian function by modulating gut microbial composition, restoring metabolic homeostasis, and suppressing inflammatory responses (Table 2). In cyclophosphamide-induced POI mouse models, intragastric administration of a mixture containing 12 probiotics for 28 days partially reversed gut and vaginal microbiota dysbiosis in POI mice. The abundances of gut-enriched genera such as *Allobaculum, Prevotella*, and *Bacteroides* were decreased, and the levels of opportunistic vaginal flora including *Proteus, Streptococcus*, and *Rothia* were also reduced. Although the improvements in hormonal profiles and follicle counts failed to reach statistical significance, the treatment group showed a tendency toward increased growing follicles and decreased atretic follicles, accompanied by remarkable alleviation of dyslipidemia (Cao et al., 2025). The abundance of *Limosilactobacillus reuteri* and its metabolite β-resorcylic acid are downregulated in cisplatin-induced POI mice, and exogenous supplementation of these substances markedly relieved chemotherapy-elicited ovarian damage (Feng et al., 2024). *Lactobacillus salivarius* LI01 can reshape gut microbiota to correct aberrant TRP metabolism, enhance ovarian antioxidant capacity and restrain excessive activation of inflammation-related pathways, thereby efficiently halting POI progression (Yao et al., 2026). *Lactiplantibacillus plantarum* P101 alleviated ovarian toxicity induced by environmental pollutants by regulating gut microbiota, reducing LPS translocation and inhibiting ovarian pyroptotic signaling (Huang et al., 2026). *Parabacteroides goldsteinii* can similarly restore ovarian reserve in mice with environmental pollutant-induced DOR, and this protective effect is linked to the amelioration of intestinal barrier impairment (Si et al., 2026).

**Table 2:** Gut microbiota-targeted interventions and their protective effects on ovarian aging and dysfunction in animal models.

Interventions		Animal models	Effects on ovarian function	Gut microbiota alterations	Molecular targets	Mechanisms	Evidence level
Probiotics	12 kinds of probiotic mixture (Cao et al., 2025)	POI mice	Primordial, follicles↑, atretic follicles↓	*Alistipes, Rodentibacter and Roseomonas*↑*, Allobaculum, Prevotella, Bacteroides, Proteus, Streptococcus* and *Rothia*↓	TC, TG↓	Mitigated vaginal microbiota disruption, alleviated lipid metabolism disorders	Animal model only
	β-resorcylic acid by *Limosilactobacillus* (Feng et al., 2024)	POI mice	FSH↓, E_2_↑; primordial and antral follicles↑, atretic follicles↓; the loss of ovarian weight↓; capability in rescuing fertilization and early embryonic development of fertilized oocytes↑	*Limosilactobacillus reuteri*, β-resorcylic acid↑	SOX7, Bax/Bcl2↓	Modulated the gut microbiota and decreased apoptosis	FMT-supported causality
	*Lactobacillus salivarius* li01 (Yao et al., 2026)	POI mice	E_2_, AMH↑, FSH↓; ovarian index↑; primary follicles and developing follicles↑	*Bacteroidetes and Desulfobacterota↑, Firmicutes, Firmicutes/Bacteroidota ratio↓, Parasutterella, Peptococcaceae unclassified*, and *Parvibacter↓, Muribaculaceae, Bacteroides* and *Alistipes↑*	IL-1β, Caspase-1, NF-κB↓; SOD, GPX4↑; Kyun, Kyat1↑; Sirt1, NAD^+^, NAMPT↑, CD38↓	Improved antioxidant function, modulated the gut microbiota and tryptophan metabolism	Receptor validated and metabolite supplementation
	Lactiplantibacillus plantarum P101 (Huang et al., 2026)	Ovarian reserve impaired rats	Atretic follicles↓, primordial follicles↑, P, AMH↑	*Clostridia↑, Elusimicrobia↓*	CAT, SOD, T-AOC↑, TNF-α, IL-6↓, IL-10↑, LPS, TLR4↓; MUC2, Ocludin↑; caspase-1, cleaved-GSDMD, cleaved-caspase11, IL-1β, IL-18↓	Improved impaired intestinal barrier, decreased inflammatory response and oxidative stress, suppressed pyroptosis	Animal model only
	Parabacteroides goldsteinii (Li et al., 2025)	DOR mice	Restored the estrous cycle pattern, AMH, E_2_↑, FSH↓; atretic follicles↓, corpora luteas↑	/	Tjp1, Ocln, Cldn1, Cldn3, Cldn4, Cldn5↑; Reg3g, Defa, Lyz1↑, Reg3g, Defa, Lyz1↑; goblet cells↑; thickness of the intestinal mucosal layer↑; LPS↓; 7-keto-lithocholic acid↑	Restored intestinal barrier integrity	FMT-supported causality, receptor validated, and metabolite rescue
Prebiotics	Alginate Oligosaccharides (Zhang et al., 2025b)	POI mice	E_2_↑, FSH, LH↓; ovarian index↑; the regularity of the estrous cycle↑; primordial, primary, and antral follicles↑, atretic follicles↓; apoptotic granulosa cells↓	*Ligilactobacillus↑, Clostridiales, Clostridiaceae, Marinifilaceae, and Clostridium_T↓*	MDA↓, CAT, SOD↑; 4-HNE, NTY, 8-OHdG and p16↓; acetic acid and total SCFAs↑	Mitigated ovarian oxidative stress and senescence, restored gut microbiota homeostasis, and regulated the microbiota-scfas axis	Animal model only
Synbiotics	Bacillus amyloliquefaciens + inulin (Liu et al., 2026)	Aged laying hens	Laying rate, egg mass↑, ovarian index↑, E_2_, FSH, LH, FSHR, LHR↑	*Bacteroidota, Firmicutes, Firmicutes/Bacteroidota↑, Lactobacillus, Akkermansia*, and *Bacteroides↑, Escherichia, Clostridium*, and *Streptococcus↓*	FAS, MTTP, PPAR-α, APOVLDL-II, VTG-II, VLDLR↑; ROS, MDA↓, T-AOC, T-SOD, CAT, GSH-Px, NRF2, NQO1, HO-1, SOD1, SOD2, CAT, GPX1↑; Claudin-1, Claudin-2↑, NF-κB, TLR4, IL-1β, IL-6 and TNF-α↓, IL-10↑; acetic acid, propionic acid, and butyric acid↑, FFAR2, FFAR3↑	Elevated the amounts of beneficial bacteria, promoted scfas, improved gut barrier function and immune states; decreased inflammatory response and oxidative stress	Animal model only
	Saccharomyces boulardii+inulin (Liu et al., 2026)	The end-stage of POI rats	E_2_, AMH↑, FSH↓	/	MDA↓, SOD↑; BMP15, KITLG↑; Bcl2↑	Inhibited oxidative stress and cell apoptosis	Animal model only
Fecal microbiota transplantation (FMT) (Li Y. et al., 2025) (Zhang et al., 2025a) (Cao et al., 2023) (Xu et al., 2022)		Sexually mature rats	E_2_↑	/	ZO-1↑, LPS, p-PI3K, p-Akt, p-NF-κB p65, TNF-α, IL-6, and IL-1β↓	Alleviated ovarian inflammation, improved colonic barrier function	FMT-supported causality and animal model only
		DOR mice	Atretic follicles↓, AMH, E_2_↑, average litter size↑	/	LPL↑, γH2AX↓; TG, TC, LDL-C↓; ROS↓, TNF-α, IL-6↓; Ocludin, ZO-1↑	Restored gut microbiota dysbiosis, and lipid metabolism disorder	FMT-supported causality and animal model only
		Laying hens	E_2_, AMH↑; ESR1, ESR2, FSHR and AMHR↑; laying rate↑	Firmicutes, Bacteroidetes↑; Lactobacillus, Enterococcus and Bacteroides↑	ZO-2, Mucin-2↑; IL-6, IL-8 and TNF-α↓; Bcl2, SIRT1↑; Bax, caspase-3, caspase-8 and caspase-9↓	Reshaped gut microbiota, repaired the intestinal barrier, and inhibited ovarian cell apoptosis	FMT-supported causality and animal model only
		Aged mice	The first litter size↑; atretic follicles↓	Bifidobacterium and Ruminococcaceae↑	IL-4↑, IFN-γ↓, p-rpS6↓; cleaved-caspase3↓, PCNA, Ki-67↑, multinucleated giant cells↓	Improved the immune microenvironment in aged ovaries, reshaped gut microbiota	FMT-supported causality and animal model only

↑, increased; ↓, decreased.

Prebiotics refer to indigestible dietary ingredients that are selectively utilized by beneficial intestinal bacteria, represented by inulin and fructooligosaccharides. They facilitate the proliferation of endogenous beneficial bacteria rather than supplementing exogenous viable microbes (Akram et al., 2024; Kaewarsar et al., 2023). Alginate oligosaccharides, a type of natural marine polysaccharide, can restore gut microbial homeostasis and increase intestinal concentrations of total SCFAs and acetate through regulation of the gut-SCFAs axis, which mitigated ovarian oxidative stress damage and cellular senescence in POI mice (Zhang et al., 2025b).

**Figure 2:**
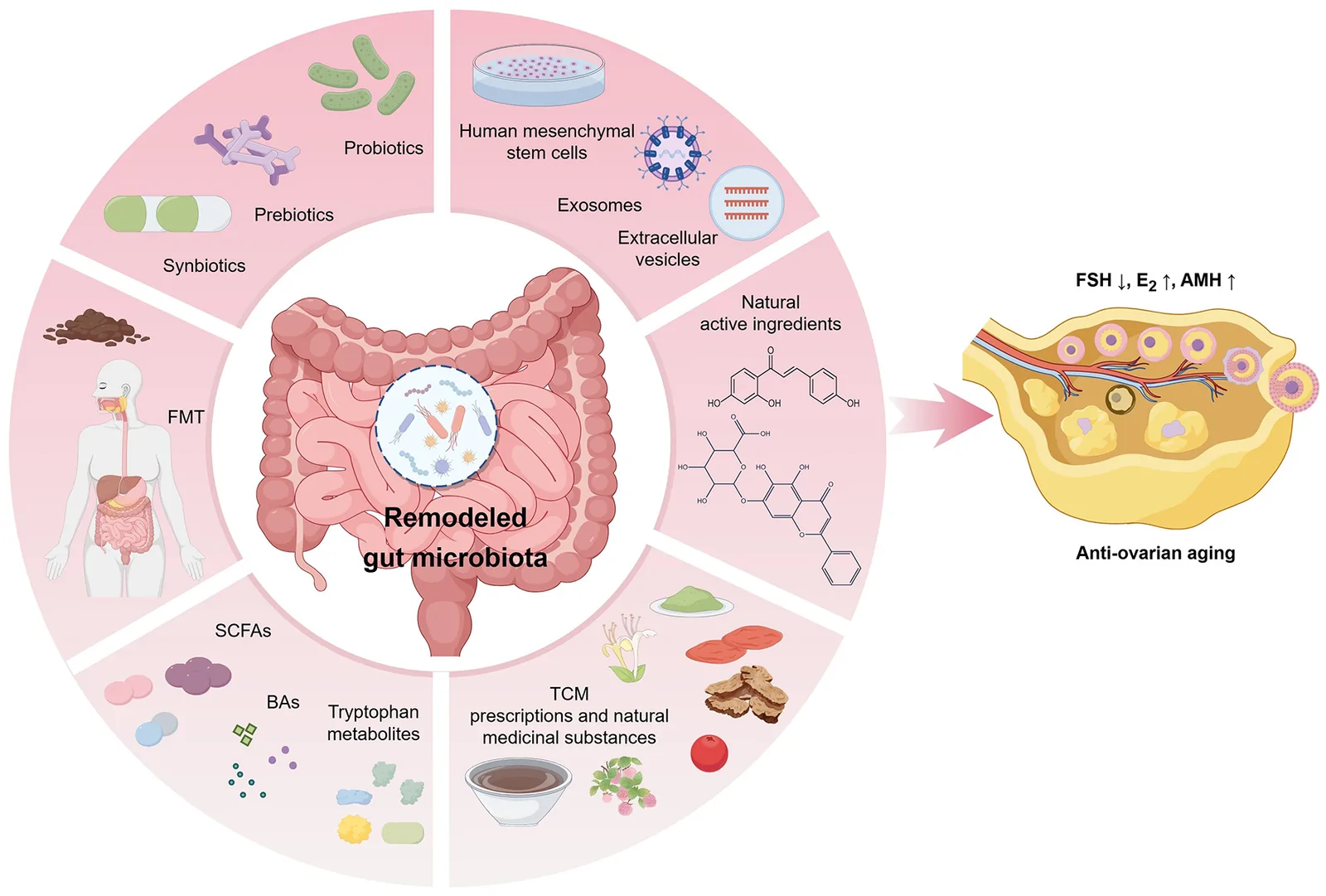
Therapeutic strategies targeting gut microbiota and related metabolites against ovarian aging. ↑, increased; ↓, decreased.

Synbiotics are combined preparations consisting of probiotics and prebiotics, which exert synergistic effects to facilitate both viable bacterial colonization and substrate-mediated microbial proliferation (Smolinska et al., 2025). Studies have demonstrated (Liu et al., 2026) that synbiotics composed of Bacillus amyloliquefaciens and inulin remodel gut microbiota in aged laying hens, elevate the abundances of SCFAs-producing beneficial bacteria including *Lactobacillus, Akkermansia*, and *Bacteroides*, enhance intestinal barrier function and immune status, alleviate ovarian oxidative stress and inflammatory responses, suppress ferroptosis-related pathways, and consequently improve egg production rate, egg weight, and egg freshness in aged laying hens. Madahali et al. (2026) systematically evaluated the therapeutic effects of *Saccharomyces boulardii*, inulin, and their corresponding synbiotic formulation in cisplatin-induced the end-stage of POI rat models. The results revealed (Madahali et al., 2026) that synbiotic intervention effectively attenuates chemotherapy-triggered oxidative stress, upregulates folliculogenesis-related genes, and downregulates pro-apoptotic gene expression, thereby facilitating follicular development, ameliorating ovarian function, and restoring hormonal profiles. Notably, synbiotics exert more pronounced synergistic effects in mitigating oxidative stress, regulating follicular maturation, and recovering endocrine function compared with single administration of either probiotics or prebiotics. Comprehensive therapeutic strategies of ovarian aging targeting gut microbiota are shown in Figure 2.

### 5.2 FMT

FMT refers to a strategy that reconstructs intestinal microecosystem via transplanting functionally active microbial communities derived from rigorously screened and processed fecal samples of healthy donors into the gastrointestinal tract of recipients. Li Y. et al. (2025) reported that transplantation of fecal microbiota from healthy donors enhances intestinal barrier function and alleviates severe deterioration of ovarian inflammatory injury and hormonal profiles via modulating the PI3K/Akt/NF-κB signaling pathway in rats co-exposed to arsenic and fluoride. Another investigation demonstrated (Zhang et al., 2025a) that FMT from healthy donors upregulates intestinal occludin and ZO-1 expression, reduces levels of reactive oxygen species (ROS), inflammatory cytokines and blood lipids, and restores oocyte quality as well as ovarian reserve in DOR model mice. Collectively, these two studies verify that FMT using healthy donor microbiota ameliorates ovarian function in animal models through reshaping gut microecology. Furthermore, transplantation of gut microbiota from breeder hens with high egg-laying efficiency directly remodels caecal microecology in low-yield broiler breeders, repairs intestinal barrier, suppresses ovarian cell apoptosis, and systematically improves ovarian function and laying performance (Cao et al., 2023). In addition, FMT derived from young healthy donors effectively reverses ovarian aging phenotypes in aged mice. Xu et al. (2022) observed that aged mice receiving fecal microbiota from young donors exhibit rejuvenated gut microbiota accompanied by elevated abundances of commensal bacteria including *Bifidobacterium* and *Ruminococcus*. Compared with non-FMT controls, FMT-treated mice display increased serum anti-inflammatory interleukin-4 (IL-4), decreased pro-inflammatory interferon-γ (IFN-γ), larger litter size of the first parity, and markedly reduced atretic follicles and apoptotic ovarian cells. Nevertheless, a recent study has yielded contradictory results. Young mice whose indigenous gut microbiota was depleted by antibiotics displayed rejuvenated ovarian transcriptomic signatures, markedly downregulated expression of inflammation-related genes, and improved ovarian health index and fertility success rate after receiving FMT from aged female mice, unexpectedly resulting in enhanced ovarian function in young recipients (Kim et al., 2026). Researchers proposed that this seemingly paradoxical phenomenon is associated with compensatory signal amplification of the estrobolome during aging. Specifically, as ovarian tissues become less responsive to gut microbial signals with advancing age, relevant microbes may exert compensatory upregulation of molecular signals; such amplified signals confer beneficial reproductive effects once transferred into young recipients (Kim et al., 2026). This study reveals the complexity and bidirectionality of crosstalk between gut microbiota and ovary, and indicates that the biological outcomes of FMT are highly dependent on gut microbial configuration and age matching between donors and recipients (Table 2).

### 5.3 Supplementation of gut microbiota-related metabolites

#### 5.3.1 SCFAs

Currently, acetate, propionate, and butyrate are the three predominant SCFAs applied in interventions against ovarian aging. Studies have demonstrated (Munyoki et al., 2025) that germ-free mice exhibit evident reproductive phenotypic defects, and SCFA intervention (67.5 mM acetate, 40 mM butyrate, 25.9 mM propionate) extends reproductive lifespan by preserving ovarian reserve. Supplementation with SCFAs also shows potential to improve reproductive performance in production practices of large mammals. *In vivo* and *in vitro* experiments conducted by Qin et al. (2025) indicated that SCFAs supplementation alleviates oxidative stress-triggered granulosa cell apoptosis and facilitates follicular development in sows. Consistently, administration of gut microbiota-derived SCFAs suppresses granulosa cell apoptosis, promotes follicular maturation and increases litter size in sows (Xu et al., 2025). Butyrate supplementation represents the strategy supported by the most abundant evidence to date. Ye et al. (2021) revealed that butyrate activates histone H3 lysine 9 (H3K9) acetylation in ovarian granulosa cells via the peroxisome proliferator-activated receptor gamma (PPARγ)/peroxisome proliferator-activated receptor gamma coactivator 1-alpha (PGC1α) pathway, restores mitochondrial dynamics and antioxidant capacity, and stimulates steroidogenesis. An *in vitro* study utilizing a three-dimensional culture system further reported that 0.10 mmol/L sodium butyrate facilitates the *in vitro* development of mouse preantral follicles and enhances oocyte quality through modulating steroidogenesis, oxidative stress, and cytoskeletal remodeling (Liu et al., 2025b) (Table 3).

**Table 3:** Gut microbiota-derived metabolites and their molecular targets and mechanisms involved in the attenuation of ovarian aging.

Interventions		Models	Effects on ovarian function	Molecular targets	Mechanisms	Evidence level
SCFAs supplementation	67.5 mM acetate, 40 mM butyrate, 25.9 mM propionate (Munyoki et al., 2025)	Germ-free mice	Primordial follicles, growing follicles↑	Nobox and Amh↑	Restored ovarian morphology	Germ-free plus rescue evidence and metabolite supplementation
	0.13% acetate, 0.11% propionate, and 0.09% butyrate (Qin et al., 2025)	Sows	Granulosa cell apoptosis↓	acetate, propionate, and butyrate↑; caspase-3↓, Bcl2↑	Alleviated apoptosis	Metabolite supplementation and pathway validated
	Propionate (Qin et al., 2025)	H_2_O_2_-induced Granulosa Cells apoptosis in Sows	Granulosa cell apoptosis↓	HDAC↓; P300↑; p-PI3K, PI3K, p-AKT, AKT↑; caspase-3↓, Bcl2↑	Alleviated apoptosis	
	41.5 mM acetate, 22.7 mM propionate, and 8.7 mM butyrate (Xu et al., 2025)	Meishan sows	Secondary follicle and antral follicle↑, atretic follicle↓; Granulosa cell apoptosis↓; litter size↑	GPR43↑, Leptin↑; LEPR, PI3K, JAK2, AKT1, AKT2, Bcl2↑	Suppressed granulosa cell apoptosis	FMT-supported causality, receptor validated, and metabolite rescue
	Butyrate (Ye et al., 2021)	Granulosa cells and KGN cells	E_2_, P↑	TC↓, H3K9ac↑, GPR43, GPR41↑, PPARγ, CD36, StAR↑, PGC1α↑, UCP2↓; CYP11A, GPR109A↓; Drp1, Fis1, Mff↓; ROS↓;	Enhanced mitochondrial dynamics and alleviated oxidative damage	Metabolite supplementation and pathway validated
	Sodium Butyrate (Liu et al., 2025b)	A three-dimensional (3D) culture system of mouse follicles	E_2_↑; the rates of follicular survival, antral formation, and ovulation↑	STAR, CYP11A1, CYP1B1↑; NRF2, SOD1↑, ROS↓; F-actin↑, GDF9, BMP15, CX37↑	Improved cytoskeletal organization and reduced oxidative stress	Metabolite supplementation
BAs intervention	LCA, NorDCA (Li et al., 2023)	Oocyte quality decline mice, Germ-free mice	Degenerated oocyte rates↓, development rates of two-cell embryos↑, blastocyst development rate↑	/	Rescued oocyte quality and early embryonic development	Germ-free plus rescue evidence, FMT-supported causality, and metabolite rescue
	TUDCA (Wang et al., 2025)	Aged porcine oocytes	Normal oocyte morphology, cortical granule distribution, and spindle structure↑; blastocyst rate and total cell number in blastocysts↑	ROS↓; GSH, SOD1↑, Caspase 3, Bax/Bcl2↓; mitochondrial membrane potential↑; GRP78, CHOP, XBP1-s against total XBP1↓	Suppressed oxidative stress, apoptosis, and endoplasmic reticulum stress	Metabolite supplementation
	TUDCA (Dicks et al., 2021)	Glucose-induced endoplasmic reticulum stress porcine embryos	Proportion of blastocyst development↑; the proportion of embryos cleavage↓	XIAP↓, CDX2, NANOG, POU5F1, SOX2, OCT4↓, SOD1, NRF2, KEAP1↑	Reduced endoplasmic reticulum stress and oxidative stress	Metabolite supplementation and pathway validated
	CDCA (Chen M. et al., 2024)	Pregnant rats	P_4_↑, embryo implantation↑, uterine receptivity↑	LIF, TGFb↑, L-methionine, L-leucine↑, TGR5, StAR, CYP11A1↑, Pgr, PR-A, Ihh↑, Ltf↓, 20α-HSD↓, p-Stat5↑, ERα↓	Drived ovarian steroidogenesis	Metabolite supplementation
	CDCA (Chen M. et al., 2024)	Pregnant sows and rats	P↑, embryo implantation↑	L-Histidine↑, TNF-α, IFN-γ, IL1β, IL6↓, T-AOC, SOD, CAT, GSH-Px↑, MDA↓, HOMA-IR↓, TC, HDL-C, LDL-C↓, glucose and insulin levels↓, HDCA, LCA, isoLCA, MDCA, UDCA, DCA, GLCA, TLCA, αMCA↑	Reduced inflammation, oxidative stress, and insulin resistance, regulated the metabolism of gut microbiota and increased the levels of secondary BAs	Metabolite supplementation and metabolite rescue
	Diets with 7.73% hyocholic acid, 68.31% hyodeoxycholic acid, and 18.96% chenodeoxycholic acid supplementation (Xing et al., 2026)	Breeder hens	Pre-grade white and yellow follicles↑	AST↓, MDA↓, TNFα↓, Claudin-1↑, IL-10↑, goblet cells↑	Reduced hepatic oxidative stress, enhanced intestinal immunity and promoted the intestinal development of offspring	Metabolite supplementation
TRY-derived metabolite supplementation	IPA (Nakayama et al., 2025)	Porcine cumulus-oocyte complexes exposed to H_2_O_2_	The rates of oocyte maturation, fertilization, and blastocyst formation↑	ROS↓, GSH↑	Enhanced oocyte quality and protecting against oxidative damage	Metabolite supplementation
	I3C (Hu et al., 2024)	Old mice	Primordial follicles↑	Nrf2, HO-1↑, LDH, MDA, ROS, and JC-1 levels↓; p-NF-κB p65, Bax and cleaved-caspase↓	Inhibited ovarian fibrosis, and attenuated ovarian apoptosis and oxidative stress	Metabolite supplementation and pathway validated
	L-kynurenine (Lu et al., 2025)	Bovine oocyte	Oocyte maturation rate and the subsequent zygote cleavage and blastocyst formation rate↑	Bax, Caspase3↓, HAS2, PTX3, and PTGS↑, GPX4, CAT, and GSH2↑, ROS↓,	Reduced apoptosis and alleviated oxidative stress	Metabolite supplementation

↑, increased; ↓, decreased.

#### 5.3.2 Selected BAs

Previous studies have predominantly linked elevated bile acid levels to adverse pregnancy outcomes. In patients with intrahepatic cholestasis of pregnancy, serum total bile acid concentrations can reach up to 40 μmol/L or even 100 μmol/L, accompanied by markedly increased levels of CA and CDCA (Huang et al., 2024; Jasak et al., 2025). Research by Zhu et al. (2025) similarly demonstrated that excessive CA accumulation is associated with suppressed ovarian steroidogenesis and impaired follicular development. Nevertheless, accumulating evidence indicates that exogenous BAs supplementation exerts moderate beneficial effects on ovarian function and pregnancy outcomes. For instance, Li et al. (2023) screened six metabolites capable of effectively rescuing oocyte quality and early embryonic development in mice from 12 gut microbial metabolites, among which LCA (30 mg/kg) and NorDCA (15 mg/kg) exhibited the most potent efficacy. TUDCA improves oocyte quality in aged pigs by alleviating oxidative stress, apoptosis and endoplasmic reticulum stress (Wang et al., 2025); additionally, TUDCA activates the TGR5 signaling pathway to attenuate high glucose-triggered endoplasmic reticulum stress and DNA damage, thereby supporting the survival and early development of porcine embryonic cells (Dicks et al., 2021). Chen M. et al. (2024) and Chen et al. (2023) reported that CDCA administration during early gestation enhances embryonic implantation in sows and rats. Xing et al. (2026) revealed that bile acid supplementation optimizes the maternal metabolic milieu, strengthens intestinal immunity and barrier function in offspring, facilitates follicular development, and confers transgenerational protective effects on ovarian function (Xing et al., 2026). Although supplementation with certain exogenous bile acids yields favorable outcomes, bile acids cannot be generally classified as beneficial supplements. Their biological effects are highly contingent on bile acid subtypes, administered dosages, receptor expression profiles, tissue microenvironments, and therapeutic time windows. Rigorous validation based on human clinical cohorts is indispensable to determine the optimal dosage, administration window and long-term safety for clinical application (Table 3).

#### 5.3.3 TRP and its derivatives

Concentrations of TRP, IPA, and IAA in follicular fluid and serum are markedly reduced in patients with DOR compared with healthy women, providing a rationale for exogenous supplementation with TRP and its derivatives (Liu et al., 2023). A randomized controlled trial enrolling 103 women undergoing IVF demonstrates that patients receiving daily oral administration of 100 mg TRP exhibit significantly elevated follicular fluid melatonin levels, together with increased numbers of retrieved oocytes and mature oocytes relative to controls. Yao et al. (2026) reported that TRP-enriched diet combined with probiotic intervention enhances ovarian antioxidant capacity and restores sex hormone levels and the number of functional follicles in POI rats via modulating gut microbiota-mediated tryptophan metabolism. Evidence suggests that IPA serves as a promising additive for *in vitro* maturation medium, with the capacity to improve porcine embryonic production efficiency by enhancing oocyte quality and counteracting oxidative injury (Nakayama et al., 2025). I3C represents another indole metabolite with protective potential against ovarian aging. Hu et al. (2024) revealed that continuous administration of 100 mg/kg I3C for 4 weeks significantly suppresses ovarian oxidative stress, cellular apoptosis and fibrosis, and increases the primordial follicle pool in 22-month-old female mice. Findings from Lu et al. (2025) indicated that L-KYN acts as a candidate antioxidant supplement for *in vitro* maturation of bovine oocytes, which improves oocyte quality and facilitates subsequent embryonic development.

### 5.4 Chinese herbal medicines

Distinct from conventional microecological interventions that directly target gut microbial composition or supplement single metabolites, Chinese herbal medicines regulate gut microbiota and ameliorate ovarian aging via unique digestive and metabolic remodeling mechanisms. At the digestive regulatory level, Chinese herbal medicines can improve intestinal peristalsis and mucosal absorption capacity, and correct gut microecological imbalance induced by disorders in substrate decomposition and nutritional metabolism. By remodeling the upstream intestinal digestive microenvironment, they indirectly facilitate the beneficial reconstruction of gut microbial community structure. At the metabolic regulatory level, herbal interventions exhibit holistic and multi-dimensional metabolic reprogramming characteristics. They comprehensively modulate metabolic networks involving glycolipid metabolism, bile acid metabolism, and amino acid metabolism, reverse gut microbiota-mediated metabolic dysfunction, and normalize the aberrant release of peripheral metabolites associated with inflammation and oxidative stress. Collectively, herbal treatments synchronously optimize intestinal digestive function, microbial structure, and systemic metabolic phenotypes, thereby establishing a stable and sustainable metabolic homeostasis *in vivo*.

#### 5.4.1 Chinese herbal formulas

Chinese herbal formulas delay ovarian aging and improve ovarian function through multiple mechanisms, including remodeling imbalanced gut microbiota and its metabolic profiles, promoting the production of beneficial metabolites such as SCFAs, repairing intestinal barrier integrity, and suppressing systemic inflammation derived from the gut. Yangjing Shugan Decoction (YJSGD) ameliorates sex hormone disorders and abnormal follicular development in the end-stage of POI mice by regulating the Sirtuin 1(Sirt1)/ Nuclear factor erythroid 2-related factor 2 (Nrf2) antioxidant pathway and the gut microbiota-metabolite axis, promoting the generation of SCFAs and reverses serum metabolic perturbations related to amino acid, lipid, and energy metabolism (Yang et al., 2026). Yang et al. (2024) demonstrated that He's Yangchao Formula (HSYC) elevates the abundances of *Akkermansia* and *Turicibacter* in the gut of aged mice, reprograms glutathione metabolism, alleviates age-related mitochondrial dysfunction, cellular apoptosis, and defective DNA damage repair, thereby exerting anti-ovarian aging effects. Additionally, HSYC improves POI by remodeling gut microbiota to facilitate glycolysis in ovarian granulosa cells (Hu R. et al., 2025). Dong et al. (2025) conducted a combined analysis of 16S rDNA sequencing and untargeted serum metabolomics, and the results show that Zichong Granules (ZC) significantly upregulate the abundance of *Lactobacillus*, restore key metabolic pathways including arachidonic acid metabolism and tryptophan metabolism, and comprehensively improve ovarian reserve, oocyte quality, and endometrial receptivity in DOR model mice.

#### 5.4.2 Natural medicinal substances and their active components

Dietary supplementation with Semen Cuscuta (SC) ameliorates ovarian aging and laying performance in aged laying hens. The underlying mechanisms include stabilizing mitochondria-associated membranes (MAMs) to inhibit ovarian granulosa cell apoptosis, remodeling gut microbiota to enhance intestinal barrier function, and mitigating systemic inflammation (Si et al., 2026). Gao et al. (2022) confirmed that crude extracts (EA) and ethyl acetate extracts (EAC) of *Dendrobium nobile* remodel gut microecology and exert antioxidant and anti-aging effects in aged mice. Flos Lonicerae and Baikal skullcap synergistically improve the laying performance of 70-week-old aged laying hens by modulating antioxidant capacity, immune function, cecal microbiota, and ovarian metabolites (Yu et al., 2025). Water extracts of Ampelopsis grossedentata (WEA) alleviate oxidative and inflammatory responses and effectively improve the reproductive performance of aged laying hens via regulating gut microbiota and the PI3K/AKT cascade reaction (Xiao et al., 2026). *Angelica sinensis* polysaccharide (ASP) (Wu et al., 2025) remodeled gut microbiota composition, reduced ovarian oxidative stress and inflammatory responses, restored serum sex hormone levels, and promoted follicular development, thereby ameliorating ovarian function in rats with chemotherapy-induced POI. *Lycium barbarum* polysaccharide (LBP) (Zheng et al., 2023) remodeled gut microbiota structure and regulated fecal metabolic profiles, particularly modulating three key metabolic pathways, including arginine biosynthesis, glycerophospholipid metabolism, and steroid hormone biosynthesis. It significantly improved ovarian reserve, endocrine function and fertility in POI mice, which enriched the evidence supporting that traditional Chinese medicine ameliorates POI by targeting gut microbiota and remodeling metabolic phenotypes. Wang et al. (2022) revealed that resveratrol (RV) corrects the abnormal shunting of tryptophan metabolism toward the kynurenine pathway by modulating gut microbiota structure, and relieves tert-butyl hydroperoxide (tBHP)-induced oxidative ovarian damage. Further FMT experiments confirm that the ovarian protective effects of RV are gut microbiota-dependent. Baicalin methyl ester (BME) alleviates reproductive injury by repairing intestinal barrier function and inhibiting the ovarian TLR4/NF-κB pathway (Liang et al., 2024). Furthermore, multiple natural products, including glycyrrhizin (GL) (Huang et al., 2025a), chlorogenic acid (CGA) (Qu et al., 2025), fisetin (FS) (Spiegel, 2025), pectin from Fructus Mori (Wang et al., 2025), and C-phycocyanin (Han et al., 2025), protect ovarian function and delay ovarian aging through multi-target synergistic effects mediated by gut microecology remodeling. These findings provide preclinical and cross-species experimental evidence for the prevention and treatment of ovarian aging. Nevertheless, their applicability to human ovarian aging remains to be further verified through rigorously designed clinical trials.

Although existing studies indicate that Chinese herbal medicines serve as promising interventions for delaying ovarian aging via gut microbiota remodeling, several limitations remain. Changes in gut microbiota observed in some studies are merely secondary therapeutic phenomena, failing to verify the direct microbiota-mediated therapeutic mechanisms. Moreover, this field faces multiple objective constraints, including insufficient quality control of medicinal materials, immature systems for active substance identification and standardized preparation, unclear multi-component and multi-target mechanisms, potential risks of drug combination, and a lack of high-quality clinical evidence in human subjects.

### 5.5 Emerging microbiota-related intervention strategies

Stem cells are a group of cells with self-renewal capacity and multi-lineage differentiation potential, which play critical roles in tissue repair, immune regulation, and regenerative medicine (Tian et al., 2023). Liu et al. (2025a) found that human placental mesenchymal stem cells systematically regulate gut microbiota composition and intestinal immune-inflammatory status, thereby alleviating POI. Xiong et al. (2025) firstly reported that Bacteroides fragilis transplantation induces intestinal epithelial cells to produce miR-1246-enriched extracellular vesicles. These vesicles are efficiently transported to the ovary via the blood circulation, target the E3 ubiquitin ligase SKP2 to reduce the ubiquitination and degradation of p62, and further activate the Kelch-like ECH-associated protein 1 (Keap1)-Nrf2 antioxidant pathway. This mechanism effectively attenuates ovarian oxidative damage and reverses reproductive aging, revealing a novel mechanism by which gut microbiota remotely regulates ovarian function via extracellular vesicle-delivered functional miRNAs. Cranberry-derived exosomes are also proven to alleviate the end-stage of POI in mice by remodeling specific gut microbiota and inhibiting granulosa cell PANoptosis (Cui et al., 2024).

Notably, although emerging strategies including stem cell therapy and microbial-derived extracellular vesicles exhibit great potential for improving ovarian function by modulating gut microecology, the microbiota-dependent specific mechanisms underlying their efficacy require further validation. Future studies should focus on elucidating the causal relationships among key microbial communities, microbial metabolites, and host signaling pathways. Clinical translational research is also essential to evaluate their safety and efficacy, so as to promote the development of innovative therapeutic strategies targeting the gut-ovary axis.

## 6 Summary and prospect

Current gut microbiota-targeted studies for ovarian aging show a progressive trend from macroscopic observation to mechanistic exploration and from basic research to clinical translation. Core intervention approaches include probiotics, prebiotics, and synbiotics. FMT enables causal verification with great translational value, microbial metabolite supplementation is an emerging novel strategy, and Chinese herbal medicine offers an innovative integrated research direction.

However, multiple challenges still exist. Most studies rely on animal models with poor human extrapolation reliability, and marked gut microbiota heterogeneity across individuals and species impedes clinical translation. Probiotic studies suffer from small sample sizes, inconsistent application standards and inadequate safety assessments. FMT is limited by unclear complex mechanisms, as well as donor screening, safety and ethical issues. Direct metabolite supplementation is restricted by short half-life and low oral bioavailability. Future research priorities include clarifying the dynamic regulatory network of gut microbiota-metabolites-ovarian function and identifying key functional microbial taxa and metabolites via multi-omics and causal analysis; conducting high-quality clinical trials and long-term follow-ups to verify the efficacy and safety of relevant interventions; and developing precise, individualized intervention strategies based on the gut-ovary axis combined with individual microbial characteristics and advanced delivery technologies.

In summary, gut microbiota-targeted interventions provide novel therapeutic insights for delaying ovarian aging. Though facing gaps between basic research and clinical application, they are promising complementary measures to conventional hormone replacement therapy and assisted reproductive technologies.
